# Genomic insights into anthropozoonotic tuberculosis in captive sun bears (*Helarctos malayanus*) and an Asiatic black bear (*Ursus thibetanus*) in Cambodia

**DOI:** 10.1038/s41598-024-57318-1

**Published:** 2024-03-28

**Authors:** Kirsty Officer, Timothy M. Walker, Sokleaph Cheng, Seiha Heng, Mallorie Hidé, Anne-Laure Bañuls, Jonathan Cracknell, Nev Broadis, Nhim Thy, Sam Abraham, Kris Warren, Bethany Jackson

**Affiliations:** 1 School of Veterinary Medicine, Murdoch University, Murdoch, WA Australia; 2 Free the Bears, Phnom Penh, Cambodia; 3 Oxford University Clinical Research Unit, Ho Chi Minh City, Vietnam; 4 Centre for Tropical Medicine and Global Health, Nuffield Department of Medicine, University of Oxford, Oxford, UK; 5 Medical Biology Laboratory, Institut Pasteur du Cambodge (IPC), Phnom Penh, Cambodia; 6 International Joint Laboratory Drug Resistance in South East Asia, IPC, Phnom Penh, Cambodia; 7 UMR MIVEGEC, IRD – CNRS, IRD Center Montpellier, Université de Montpellier, Montpellier, France; 8 Knowsley Safari, Prescot, Merseyside, UK; 9 Forestry Administration, Ministry of Agriculture, Forestry and Fisheries, Phnom Penh, Cambodia; 10 Antimicrobial Resistance and Infectious Diseases Laboratory, Harry Butler Institute, Murdoch University, Murdoch, WA Australia; 11 Centre for Terrestrial Ecosystem Science and Sustainability, Harry Butler Institute, Murdoch University, Murdoch, WA Australia

**Keywords:** Tuberculosis, Zoology, Pathogens

## Abstract

Contact between humans and wildlife presents a risk for both zoonotic and anthropozoonotic disease transmission. In this study we report the detection of human strains of *Mycobacterium tuberculosis* in sun bears and an Asiatic black bear in a wildlife rescue centre in Cambodia, confirming for the first time the susceptibility of these bear species to tuberculosis when in close contact with humans. After genotyping revealed two different strains of *M. tuberculosis* from cases occurring between 2009 and 2019, 100 isolates from 30 sun bear cases, a single Asiatic black bear case, and a human case were subjected to whole genome sequencing. We combined single nucleotide polymorphism analysis and exploration of mixed base calls with epidemiological data to indicate the evolution of each outbreak. Our results confirmed two concurrent yet separate tuberculosis outbreaks and established a likely transmission route in one outbreak where the human case acted as an intermediatory between bear cases. In both outbreaks, we observed high rates of transmission and progression to active disease, suggesting that sun bears are highly susceptible to tuberculosis if exposed under these conditions. Overall, our findings highlight the risk of bi-directional transmission of tuberculosis between humans and captive bears in high human tuberculosis burden regions, with implied considerations for veterinary and public health. We also demonstrate the use of standard genomic approaches to better understand disease outbreaks in captive wildlife settings and to inform control and prevention measures.

## Introduction

Tuberculosis (TB) is a global One Health challenge, affecting humans, domestic animals, and wildlife. With an estimated one quarter of the world’s population infected at some time, and 1.5 million human deaths annually, TB remains the most impactful bacterial disease globally^1^. The impact is disproportionately felt, with TB intrinsically linked to poverty, and perpetuated by high population densities and barriers to healthcare access^2^. As a country with high TB caseloads, Cambodia remains on the United Nations global TB watchlist^1^, with control hampered by an estimated 34% of cases that go undiagnosed and unreported^3^. In this and similar regions of high TB burden, human-wildlife interfaces increase the likelihood of TB spillover to, and back from, previously unreported species, increasing the need for effective surveillance to protect both wildlife and public health.

Tuberculosis in humans and other animals is caused by members of the *Mycobacterium tuberculosis* complex (MTBC). In humans, TB is primarily caused by *Mycobacterium tuberculosis* sensu stricto, while several other members of the MTBC are animal-adapted, with specific livestock and wildlife host preferences^4^. Spill-over to non-host species is relatively common for the animal-adapted MTBC species and for some, wildlife populations have created reservoirs of infection with concomitant challenges for control in the livestock host^5–7^. In contrast, *M. tuberculosis* is generally considered an obligate human pathogen, with human hosts needed to maintain infection and transmission cycles^8^. As such, non-human host populations may be limited in their ability to sustain infection without ongoing exposure^9–11^. However, since the 1990s M*. tuberculosis* has emerged and persisted in elephants, with evidence of spread between elephants and to other species, including back to humans^12–23^. The emergence of *M. tuberculosis* in elephants, and occasional cases in other, often primate, wildlife species^24–35^, is likely due to amplification of human-wildlife contact combined with improved diagnostics, surveillance, and reporting.

In *M. tuberculosis* spill-over events, genotyping and whole genome sequencing (WGS) methods have proven useful in demonstrating inter- and intra-species transmission, as well as supporting prevention and management strategies^12,13,21,29,36^. In high human TB burden regions, laboratory services are often necessarily focused on diagnosis and identification of key antimicrobial resistance patterns to enable the rapid initiation of appropriate treatment^37^. WGS is not always practical or routinely accessible, thereby limiting its availability for incidental veterinary investigations.

There are occasional reports of *M. tuberculosis* infection in captive bears^38–41^, most commonly sloth bears (*Melursus ursinus*) rescued from exploitative entertainment industries which historically put them in prolonged close contact with humans^42–44^. In this study we report for the first time *M. tuberculosis* infection causing TB in sun bears (*Helarctos malayanus*) and an Asiatic black bear (*Ursus thibetanus*), at a bear rescue facility in Cambodia. To further investigate the outbreak epidemiology, we conducted whole genome sequencing on *M. tuberculosis* isolates from bear cases, and from a human case with apparent occupational exposure.

## Results

### *Mycobacterium tuberculosis* cases

**Figure 1:**
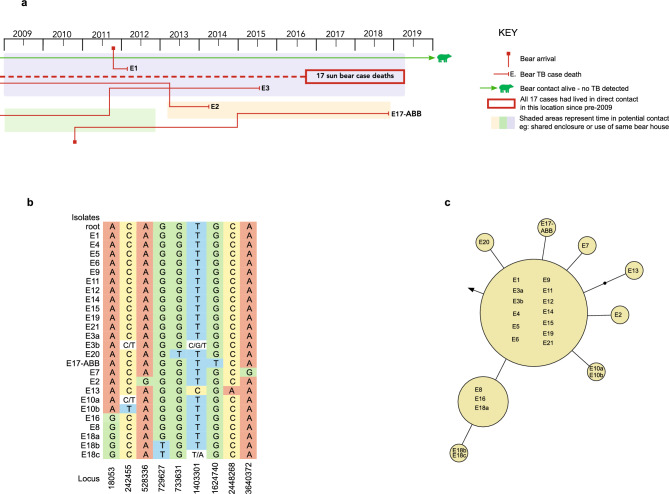
Detailed review of the Lineage 1 *Mycobacterium tuberculosis* outbreak. Spatiotemporal mapping of cases (**a**). Matrix of nucleotide variants (**b**). Case numbers E1 – E21 are shown, with the isolates reference representing isolates from each case with no nucleotide variants, or with a lower-case letter used to distinguish between isolates from the same case that had nucleotide variants (see also Table 2). Phylogenetic topology of isolates, with genetic distances estimated with maximum likelihood (**c**). Each yellow circle represents a node of isolates separated by no single nucleotide polymorphisms (SNPs); a line connecting two yellow circles represents a difference of one SNP; a solid black circle is added to represent each additional SNP difference. The arrow indicates the position of the root of the tree.

**Figure 2:**
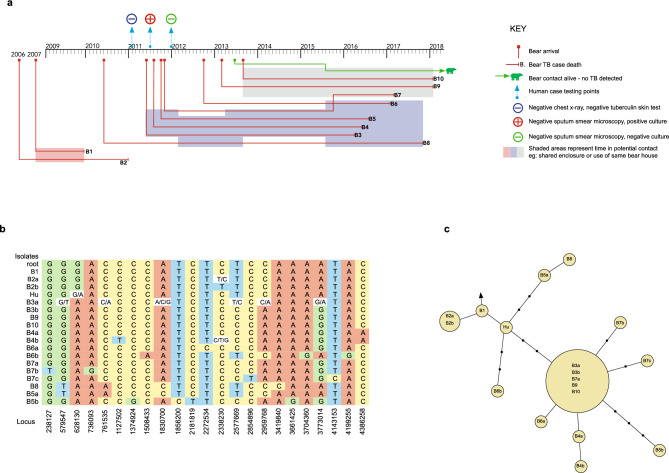
Detailed review of the Lineage 2 *Mycobacterium tuberculosis* outbreak. Spatiotemporal mapping of cases (**a**). Matrix of nucleotide variants (**b**). Bear cases B1–B10, and a human case (Hu) are shown, with the isolates reference representing isolates from each case with no nucleotide variants, or with a lower-case letter used to distinguish between isolates from the same case that had nucleotide variants (see also Table 1). Phylogenetic topology of isolates, with genetic distances estimated with maximum likelihood (**c**). Each yellow circle represents a node of isolates separated by no single nucleotide polymorphisms (SNPs); a line connecting two yellow circles represents a difference of one SNP; a solid black circle is added to represent each additional SNP difference. The arrow indicates the position of the root of the tree.

Between 2009 and 2019 the bear population at the Cambodia Bear Sanctuary (CBS) ranged from 102 to 136 bears at any one time (75 to 98 sun bears; 27 to 42 Asiatic black bears) with a cumulative total over the study period of 183 bears (132 sun bears; 51 Asiatic black bears) at risk. A total of 30 sun bears, one Asiatic black bear, and a member of staff were culture positive for *M. tuberculosis* on at least one diagnostic sample. The human case fully recovered following treatment, and no further staff cases were declared during the study period. All bear cases with *M. tuberculosis* confirmed by culture either died naturally (n = 3), died under anaesthetic (n = 1), or were humanely euthanized (n = 27). All except seven bear cases were showing clinical signs consistent with TB at the time of diagnosis. Treatment of bears with human anti-tuberculosis medication was not permitted and therefore not attempted for any cases. All bear cases had signs of TB on postmortem examination, ranging from lymph node enlargement to advanced pulmonary disease and dissemination to other organs. Sixteen bear cases had evidence of extrapulmonary disease, including four with no evidence of pulmonary disease and with *M. tuberculosis* cultured from non-pulmonary sites only. Most bear cases were clustered around two sites within the sanctuary, with 17 cases occurring in a single bear group between August 2016 and March 2019 (Fig. 1a), and eight cases occurring in two neighbouring groups in a single bear house between February 2016 and February 2018 (Fig. 2a).

### Phenotypic antimicrobial susceptibility testing

Phenotypic antimicrobial susceptibility testing results are shown in Table S1, which also summarises the species, age, and arrival date of each case. Susceptibility testing using one diagnostic sample from each case revealed two patterns. Isolates from eleven cases showed resistance to isoniazid and streptomycin, whereas isolates from 21 cases were sensitive to all four anti-tuberculosis drugs tested.

### Genotyping

Spoligotyping and MIRU-VNTR typing results are included in Supplementary Material 1. The genotyping results were consistent with all cases being infected with human *M. tuberculosis* belonging to either the East African-Indian or Beijing spoligotype families. These results confirmed two different infection chains as suggested by the two antimicrobial susceptibility patterns and motivated the subsequent whole genome sequencing of all available isolates.

### Whole genome sequencing

A total of 102 isolates originating from 31 bear TB cases and one human TB case were available for whole genome sequencing (WGS). For 26 of the 31 bear cases, multiple isolates were available due to specimens collected from different anatomical sites or via different sampling techniques. For the remaining five bear cases and the human case, *M. tuberculosis* was cultured from one specimen only. Of the 102 isolates that underwent WGS, 100 were successfully sequenced and had sufficient data available for inclusion in the analysis. Sequence analysis confirmed that all 100 isolates were human-adapted strains of *M. tuberculosis*. Sixty-nine isolates from 20 sun bears and one Asiatic black bear belonged to *M. tuberculosis* Lineage 1 (Indo-Oceanic), and 31 isolates from ten sun bears and a human belonged to Lineage 2 (East Asian/Beijing) (Table S2). Sequence data supported the phenotypic susceptibility testing results, with the 40 lineage 2 isolates showing the *fabG1* C-15 T mutation causing isoniazid resistance, along with the *rpsL* K88R mutation causing streptomycin resistance. None of the lineage 1 isolates had either of these mutations, and no other resistance mutations were present on WGS.

### Anatomical sites of isolation

The sites of origin of isolates are summarised in Tables 1 and 2. Isolates from the same case with genetic variation are distinguished by a letter after the case ID as shown in the tables and the same nomenclature is used in the SNP matrices and phylogenetic topology in Figs. 1 and 2. Sample sites are broadly categorised as pulmonary or extra-pulmonary, with positive cultures from gastric fluid or faeces, in the absence of any macroscopic evidence of extra-pulmonary disease, considered to be of pulmonary origin^45,46^. *M. tuberculosis* was cultured from extra-pulmonary sites in 7/10 (70%) Lineage 2 cases, including three cases with no evidence of pulmonary disease, whereas in Lineage 1 M*. tuberculosis* was cultured from extra-pulmonary sites in 9/21 (43%) cases..

**Table 1:** Sampling date and origin of Lineage 1 isolates from tuberculosis cases in sun bears (*Helarctos malayanus*) and an Asiatic black bear (*Ursus thibetanus*) at the Cambodia Bear Sanctuary subjected to whole genome sequencing.

Case ID	Postmortem date	Isolates reference^	Pulmonary origin	Extra-pulmonary origin
E1	Dec 2011	E1	BAL	–
E2	Mar 2014	E2	BAL	–
E3	Jul 2015	E3a	Lung 1, pleural fluid	–
		E3b	Lung 2	–
E4	Aug 2016	E4	BAL, lung	–
E5	Nov 2016	E5	BAL, lung, faeces*	–
E6	Nov 2016	E6	Lung, faeces	–
E7	Mar 2017	E7	BAL, lung, tracheal mucous, pleural fluid, faeces*	–
E8	Mar 2017	E8	BAL, lung, faeces*	–
E9	Mar 2017	E9	Lung	–
E10	Mar 2017	E10a	Lung	–
		E10b	BAL	–
E11	Mar 2017	E11	Lung, faeces*	–
E12	Mar 2017	E12	Lung	–
E13	Nov 2017	E13	BAL, lung	Tarsal joint cartilage, (tracheobronchial LN)**
E14	Nov 2017	E14	BAL, lung	Tracheobronchial LN, mediastinal LN, liver
E15	Dec 2017	E15	BAL, lung (× 2), pleural fluid	Tracheobronchial LN, mesenteric LN
E16	Aug 2018	E16	–	NHW (× 3)^#^,mediastinal LN, (mesenteric LN)**
E17-ABB	Nov 2018	E17-ABB	BAL, lung	Liver
E18	Nov 2018	E18a	BAL, lung	Mediastinal LN, tracheobronchial LN, mesenteric LN
		E18b	–	Prescapular LN
		E18c	–	Prescapular abscess
E19	Dec 2018	E19	BAL, lung	Tracheobronchial LN, mesenteric LN, peritoneal fluid
E20	Dec 2018	E20	Lung	Mediastinal LN, spleen, liver, faeces
E21	Mar 2019	E21	BAL, lung	Submandibular LN, mediastinal LN, mesenteric LN

BAL: bronchoalveolar lavage, LN: lymph node, NHW: non healing wound.

^Isolates from each case with no genetic variation are grouped together and referred to by the case ID and a lower-case letter.

*Gastrointestinal content samples are considered pulmonary when there was no macroscopic evidence of extra-pulmonary disease on postmortem examination.

^#^two wound samples were collected five and three months before remaining samples.

**isolate failed sequencing.

**Table 2:** Sampling date and origin of 31 Lineage 2 isolates from tuberculosis cases in sun bears (*Helarctos malayanus*) and a human at the Cambodia Bear Sanctuary subjected to whole genome sequencing.

Case ID	Postmortem date	Isolates reference^	Pulmonary origin	Extra-pulmonary origin
B1	Dec 2009	B1	BAL, lung, gastric fluid*	–
B2	Jan 2011	B2a	Lung	–
		B2b	Lung (×2), pleural fluid	–
Hu	Jul 2011	Hu	Sputum	–
B3	Feb 2016	B3a	Tracheal mucous	–
		B3b	BAL	–
B4	Jul 2016	B4a	BAL	NHW × 2, oral swab
		B4b	Lung	–
B5	Aug 2016	B5a	Lung	–
		B5b	–	Cervical LN, oral biopsy
B6	Feb 2017	B6a	Lung	Peritoneal fluid, SC tissue
		B6b	–	Intestinal abscess
B7	Mar 2017	B7a	–	Mesenteric LN
		B7b	–	Tongue lesion, small intestine
		B7c	Lung	–
B8	Nov 2017	B8	–	Submandibular LN, tracheobronchial LN
B9	Feb 2018	B9	–	Submandibular LN
B10	Feb 2018	B10	–	Tracheobronchial LN, mesenteric LN

BAL: bronchoalveolar lavage, NHW: non healing wound, LN: lymph node, SC: sub-cutaneous.

^Isolates from each case with no genetic variation are grouped together and referred to by the case ID and a lower-case letter.

*Gastrointestinal content samples are considered pulmonary when there was no macroscopic evidence of extra-pulmonary disease on postmortem examination.

### Genomic diversity of *M. tuberculosis* isolates

Phylogenetic analysis demonstrated two genomic clusters, one of Lineage 1 and one of Lineage 2, with 1700 SNPs separating the outbreaks, and high genomic similarity within each cluster. Of the 69 Lineage 1 isolates, 36 isolates from 12 bear cases showed zero SNP differences with respect to one another. The distance between any two of the remaining 33 isolates ranged from zero to four SNPs, with no two isolates from different bear cases separated by more than three SNPs (Fig. 1c). Of the 31 Lineage 2 isolates, inter-host distances ranged from zero to 11 SNPs, with the highest minimum distance between cases being 8 SNPs (Fig. 2c). The number of isolates sequenced from each case ranged from one to seven, with more than one isolate available for 26 cases (Tables 1 and 2). For 21 of 26 cases there was zero SNP difference between intra-host isolates. For the five cases with intra-host SNP diversity (one Lineage 1, four Lineage 2 cases), SNP differences ranged from one to ten SNPs. Thirteen variant loci with evidence of more than one allele were identified, including six in one isolate (B3’s tracheal isolate: B3a).

### Correlation with spatiotemporal data

Genomic data confirmed key transmission links that were suspected through mapping of cases and genotyping. Figures 1 and 2 provide spatiotemporal data on the outbreaks (1a/2a), SNP matrices examining inter- and intra-host isolate variations at the allelic level (1b/2b), and the maximum likelihood phylogenetic topography for each outbreak (1c/2c). Taken together, these results provide molecular epidemiological insights for each outbreak.

The Lineage 1 outbreak was centered around one bear house (Fig. 1a). In late 2011, 20 of the 21 bears diagnosed with TB were living in the same enclosure or in indirect contact through adjoining enclosures and night dens. The phylogeny suggests a point source in this outbreak with a central node of 12 cases and the remaining isolates all within two SNPs. The most tenuous epidemiological link was for E17-ABB who developed TB in late 2018. Although E17-ABB lived in the vicinity (neighboring enclosure) of E3 seven years earlier, this pre-dated the arrival at the sanctuary of the bear (E1) who went on to become the first detected case in this outbreak.

For the Lineage 2 outbreak (Fig. 2), the first two cases were in contact (B1, B2), and within one SNP of each other. Five years separated case B2 and the next bear case B3, with B3 arriving at the sanctuary after the death of B1 and B2. However the human case (Hu), whose isolate was just one SNP from B1, did have contact with B1 and B2, and developed symptoms in mid 2011 while providing close care to B3 who had just arrived as an orphaned cub. On routine staff testing six months previously this individual showed no symptoms, had no evidence of TB on chest radiography, and had a negative tuberculin skin test. After B3’s death four and a half years later, seven more cases followed. Of these, five had direct contact with B3, with a clear transmission path to the final two cases (B9 and B10) via B7.

Based on the epidemiology and the phylogeny, we inferred that the Lineage 2 outbreak likely started with bear cases B1 and B2, and that the human case was an intermediary between these and the other bear cases. The sequence of B1’s isolate matched the root (Fig. 2b), making it likely that transmission occurred from the human population to this bear, although a lack of testing and clinical records prior to 2009 meant we were unable to look further into infection sources for this case. Bears with possible exposure to B1 and B2 have undergone regular TB screening and until the time of publication none have developed clinical signs or tested positive for *M. tuberculosis*, making it unlikely that an undetected case, rather than the human case, formed the link between B1 and/or B2 and the next bear case, B3. To further test our hypothesis that transmission in this outbreak then occurred from the human case to B3 and on to the remaining bear cases, we inspected the Variant Call Format (VCF) files at loci across other isolates with null calls for evidence of two alleles (we chose to disregard null calls masking three alleles as the signal to noise ratio here was assumed to be less favourable). The one null call identified from the human case was at locus 628130 where there was evidence of both Guanine (‘G’), reflecting the allele seen in B1 and B2, but also of Adenine (‘A’), which was found at this locus in all other subsequent cases (Fig. 2b). This was consistent with the human as the intermediary case. However, whilst the phylogeny is suggestive of the human also as the source for B5a, B8, and B6b (Fig. 2c), it was null calls in B3’s tracheal isolate strain (B3a) rather than any in the human strain that masked the presence of alleles distinguishing at least isolates B5a and B8 from the human strain. At locus 579547 only B3a shows both G and Thymine (‘T’), where G is present in all other strains, apart from isolates B5a and B8 where T is seen. Likewise, at locus 761535 B3a has both Cytosine (‘C’) and A, where C is present in all other strains, apart from isolates B5a and B8 where A is seen. These findings are more consistent with B3 having infected B5 and B8, whereas the phylogeny indicates that the human case was more likely the source. The epidemiological data also supports B3 as the source of infection to B5 and B8, with both bears being in direct contact with B3, and neither bear diagnosed nor developing signs of disease until after B3’s death with advanced TB (Fig. 2a). Similarly, the phylogenetic tree is consistent with transmission from the human to B6b, rather than from B3 (Fig. 2c). However, manual inspection of the FASTA file of variants (Fig. 2b) revealed that the additional three SNP difference between B6b and B3a seen in the tree is the result of imputation by the maximum likelihood tree building method because of null calls at the variant sites. By inspecting the VCF files manually to understand the reason for these null calls we found evidence for more than one allele, with the alleles seen in the human strain and B6b both reflected in the alternative alleles seen in B3a. With the diversity seen in B3a’s sequence reflected in both Hu and B6b’s sequences, it is therefore just as possible that B3, rather than the human, infected B6 directly. This was supported by the contact data which saw B6 arrive well after resolution of the human case, and live in close contact with B3 when that bear developed active disease (Fig. 2a). The SNP matrix, phylogenetic information, and contact data were all consistent with B3 being the source of infection of the remaining bears.

## Discussion

This study used WGS to confirm two concurrent yet separate outbreaks of human TB lineages involving captive sun bears and an Asiatic black bear at a bear sanctuary in Cambodia. To our knowledge this is the first report confirming *M. tuberculosis* infection in either species, and the findings highlight the potential risks to public health and veterinary disease control for captive bear facilities in high human TB regions. WGS results allowed us to infer transmission dynamics and to confirm the involvement of a human case, thereby demonstrating the potential for bidirectional transmission of *M. tuberculosis* between humans and sun bears under these conditions. We suggest sun bears are highly susceptible to infection with human adapted *M. tuberculosis* lineages, with transmission between sun bears in close proximity allowing infection to be maintained for more than seven years in each outbreak, and progression to culture-confirmed active disease seen in all but one sun bear in each of the highly exposed groups at the centre of each outbreak.

A combination of epidemiological and genomic data was used to reconstruct the evolution of each outbreak, paying particular attention to the information hidden within null calls within the multi-FASTA alignment of variant sites. The Lineage 2 outbreak followed more of a linear pattern with successive hosts appearing to pass the infection on, whereas the Lineage 1 outbreak was more consistent with a point source outbreak given the star-like phylogenetic topology. For all but one of the null calls masking just two alleles in either outbreak, those alleles were seen as variants at the relevant locus in other outbreak strains, underlining the potential for these sites to be informative about direction of transmission. However, these data need to be understood in the context of the timing of sampling. Although one mixed call in the human case of the Lineage 2 outbreak supported that case being an intermediary between two bears, the additional loci with mixed calls seen in isolate B3a were less consistent with the phylogeny, making more sense if this isolate was seen at the node in the phylogenetic tree where the human strain was placed. One possible explanation is that B3's strain did at one point reflect the expected sequence at that node, but that by the time samples were taken from the bear, three SNPs had evolved, placing those strains at a distance from the human. Case B3 may therefore have been the source for B5a and B6b, rather than the human, as the phylogeny would otherwise suggest. For this outbreak, combining the phylogenetic information with analysis of the null calls revealed a plausible transmission route that was consistent with the epidemiological data and thereby demonstrating likely bidirectional transmission between human and sun bear cases.

In each outbreak, only one bear in contact with multiple cases did not develop TB. Considering the relatively well-ventilated environment, with bears spending most of their time in outdoor enclosures, the ease of transmission and disease progression in almost all close contacts within the outbreak groups is concerning and has consequences for TB control in this and similar settings. This finding contrasts with data from human households, which reveal that around 50% of close contacts with prolonged exposure to TB cases become latently infected, and less than 5% go on to develop active TB^47^. A lack of spread among in-contact conspecifics in zoo environments has also been shown^40,48^, although group-housed elephants and primates have successfully propagated single strains of *M. tuberculosis* over time^12,13,29^. The advanced pulmonary disease seen in most bears cases at diagnosis, along with exposure to multiple contacts, plausibly contributed to the ease of spread, through increased exposure to highly infectious aerosols for individuals in proximity. Our confirmation of disease in almost all bears in two close contact groups, with most detected within a two-year period for each group, suggests that clearance or life-long immune system containment is rare in sun bears under these conditions. In contrast, only a small proportion of all humans infected with *M. tuberculosis* go on to develop disease in their lifetime^49^, and long-term serological studies in Asian elephants suggest latent infection may occur in this species^16,19,50–53^. It is likely that the response to a TB incursion within a given environment is driven by idiosyncratic relationships within the host-environment-agent paradigm, and it is possible that stressors related to captivity affect the immunocompetence of exposed bears to enhance the likelihood of infection, disease progression, and effective transmission.

Although no human source was identified, detection of the two dominant TB lineages in the human population of Cambodia (Lineage 1 (Indo-Oceanic) and 2 (East Asian/Beijing))^54,55^, as well as drug resistance (no bears had been exposed to anti-TB drugs), support both bear outbreaks being of human origin. Case E1’s history of living within a city household, along with disease onset so soon after arrival, suggests this bear was the index case in the Lineage 1 outbreak, and highlights the risk to bear sanctuaries receiving bears with a history of co-habiting with humans. Given the anthropozoonotic spread of TB in other species with human contact^17,20,42^, along with the high numbers of sun bears and Asiatic black bears held in captivity (legally and illegally) in high TB burden regions^56–60^, it is surprising this is the first report of incidental spill-over from humans to these species. Regardless, our findings confirm that both bear species are susceptible to human lineages of *M. tuberculosis* and should inform sanctuary disease screening strategies. Genomic evidence of spread to one bear (E17-ABB) with no known direct contact with an active case, along with confirmation of mycobacterial shedding from the gastrointestinal tract and superficial wounds, reinforces the possibility that, apart from respiratory exposure, fomites (such as bedding, tools, and veterinary equipment) constitute a potential transmission risk, as highlighted elsewhere^21^. Testing for subclinical TB is inherently insensitive^61,62^, and the resulting probability of false negative results raises the possibility that one or more undetected cases within the sanctuary formed links between known bear cases. While this cannot be ruled out, it is considered unlikely in these bear outbreaks. When TB becomes active, and thereby transmissible, accelerated mycobacterial replication increases the likelihood of pathological changes, immunological responses, clinical signs, and positive test results^63^. Given the ongoing close observation and regular testing of bears at the sanctuary, the likelihood that an active case transmitted infection yet remained healthy and negative on all testing to the time of publication (over five years and eight years after the deaths of E17-ABB and B3, respectively) is low. Nevertheless, the ability for mycobacterial infections to remain virtually undetectable until disease is active and progressing is a critical consideration, and we therefore specifically recommend screening protocols are judiciously applied to potentially exposed bears on a regular and ongoing basis to optimise detection before onward transmission has occurred.

The level of intra-host SNP accumulation (0-10 SNPs) seen was similar to what has previously been observed in humans^64^ and in cross-sectional isolates from elephants^12^. Microevolution was seen between isolates from pulmonary tract samples collected from the same patient on the same day, reinforcing the importance of multi-site sampling, even within the respiratory tract, if genomic data is used to explore microevolution and make transmission inferences.

This study uniquely confirms that sun bears and Asiatic black bears are susceptible spill-over hosts for *M. tuberculosis*, with a high risk of developing active TB in a captive environment. The implied zoonotic and anthropozoonotic risks associated with human TB strains in captive bears has clear public, occupational, and veterinary health ramifications for sanctuaries in areas of high human TB burdens. Given the multiple threats already faced by bears in the region, these findings have conservation implications and demonstrate the need for TB to be considered if captive individuals are regarded for future release and repopulation strategies. The study draws attention to the utility of standard genomic approaches in captive wildlife outbreaks and their potential to complement epidemiological investigations to enhance the effective and timely implementation of control and prevention measures. Our findings add to calls for TB risk management to explicitly address areas of human-wildlife interaction in TB endemic regions^17,23^, and to be enhanced by more accessible genomic methods, ideally through locally embedded and synergistically linked human and animal laboratory services.

## Methods

### Ethics declaration

This study involved the opportunistic use of animal samples collected and archived during veterinary procedures and postmortem examinations originally carried out to investigate a disease outbreak. No animals were anaesthetised or euthanized for the specific purpose of this study, with all samples originally collected using standard veterinary techniques and procedures for clinical and pathological examinations. Ethics approval for the use of animal samples in this project was obtained from Murdoch University (Animal Ethics permit number R3276/20), and all work was carried out in accordance with permit guidelines. The study is reported in accordance with ARRIVE guidelines (https://arriveguidelines.org). Ethics approval for the use of genetic material from a single human diagnostic sample was obtained from Murdoch University (Human Ethics permit number 2020/159) and all work was carried out in accordance with permit guidelines, including acquisition of informed written consent from the human participant. Approval to transfer inactivated *M. tuberculosis* isolates from Cambodia to Australia was obtained via BICON permit number IP0004857803 for the importation of biological materials.

### Study location and background

This study investigates an outbreak of tuberculosis (TB) at the Cambodia Bear Sanctuary (CBS), (1° 18′ 06.5″ N, 104° 48′ 04.7″ E). The bear population at the CBS is dynamic, with intermittent receivals of sun bears and Asiatic black bears intercepted by the Forestry Administration of Cambodia from illegal situations including poaching, trade, and keeping of these protected species. Bears are provided with life-long care by a local husbandry team, supported by international bear welfare and conservation organisation Free the Bears. The welfare of bears at the sanctuary is closely monitored with twice daily visual observations and supported by an on-site veterinary team and purpose-built veterinary hospital. Written husbandry and veterinary protocols guide the daily care, nutrition, and reporting and investigation of any health concerns. Re-release to the wild is not currently undertaken, however it is an active long-term goal for the conservation of bear species in Cambodia. The sanctuary is spread over a 100 hectare site and consists of eight bear houses. Each house has between two and six outdoor enclosures attached, with a total of 28 enclosures ranging in area from approximately 500 to 8000 square metres. The number and unpredictable nature of bear receivals creates demand for limited space within the sanctuary, and group housing along with movement of individuals or groups between enclosures are often required for management purposes. Along with the outdoor enclosures, bears have access to indoor well-ventilated dens within bear houses, and contact between groups of bears is possible through bars between dens and/or adjoining fences between enclosures.

### Study design

This study uses a clinical case series to retrospectively investigate the molecular epidemiology of a disease outbreak and as such the inclusion of control subjects, the randomisation of animals or samples, and the blinding of investigators was not undertaken. The sample size was determined by the number of cases. We defined a case as any species with bacteriologically confirmed TB at the CBS between 2009 and 2019, whether human, sun bear, or Asiatic black bear. All confirmed TB cases were included in the investigation.

### Outbreak investigation

After an outbreak of TB was first suspected, investigation began with a review of the sanctuary’s husbandry and veterinary records to determine the movements of confirmed cases within the sanctuary, along with identification of bears with a history of direct contact (living in the same group) or potential contact (living in a neighbouring group) with cases. An ongoing strategy of routine screening for active cases was initiated, prioritising bears based on the duration and intensity of contact with confirmed cases, along with testing of any bears showing clinical signs consistent with TB. Staff and work rosters were consulted to investigate potential contacts, results of historical ad-hoc staff TB testing were collated, and a program of yearly chest radiographs of all staff was commenced. After TB cases were confirmed by culture, spatial and contact data were aligned to details of isolates to further characterise the outbreak. These details included antimicrobial susceptibility patterns, genotyping, and finally whole genome sequencing.

### Sample collection

Depending on the case, samples originating from bears were collected antemortem and/or postmortem. Antemortem sampling was either through diagnostic work up of bears showing clinical signs consistent with active TB (including unexplained weight loss, inappetence, lethargy, respiratory signs, lymphadenopathy, or presence of a non-healing wound) or through screening of in-contact/at risk bears. Any bear with TB confirmed on antemortem testing was humanely euthanised. All antemortem samples were collected within five months of death and postmortem. Sample types included bronchoalveolar lavage (BAL), wound biopsy, faeces, oral swab and biopsy, fluid aspirate, and postmortem tissue and fluid sampling. Apart from faeces, all antemortem samples were collected under general anaesthesia. Bears were immobilised via intra-muscular blow dart (Daninject, Australia) with medetomidine (Medetomidine, Troy Laboratories, New Zealand) and zolazepam/tiletamine (Zoletil, Virbac, Australia), administered at a dose rate of 0.0125 mg/kg medetomidine and 1.25 mg/kg zolazepam/tiletamine. After intubation, anaesthesia was maintained with isoflurane (Forane, Baxter Healthcare, USA) and oxygen delivered via a circle re-breathing circuit (CycloFlo, Burton’s Medical Equipment, UK). BAL samples were collected by passing a sterile 10–12F feeding tube through the endotracheal tube until resistance was felt, indicating the level of the lower airways (usually 70–90 cm depending on the size of the bear). A volume of 60–200 ml of sterile saline was introduced to the lung by syringe via the tube, followed by re-aspiration with the syringe while an assistant provided gentle rolling of the bear’s chest. Biopsies were collected in a sterile manner using a scalpel blade to remove a small sample of non-healing wound or oral tissue. Oral swabs were collected using a sterile cotton swab, and tracheal mucous was collected from the endotracheal tube on extubation. One peritoneal fluid sample was collected antemortem by introducing a sterile 16 gauge 51 mm intravenous catheter through an area of aseptically prepared skin on the ventral abdomen. If tissue biopsies were performed analgesia was provided using meloxicam (Mobic, Boehringer-Ingelheim, USA) given subcutaneously (0.2 mg/kg) at the time of biopsy and orally (0.1 mg/kg) in the following days if deemed necessary. Euthanasia was performed under general anesthesia using pentobarbitone sodium solution (Virbac Animal Health, Australia) injected intravenously with death confirmed by detection of the cessation of an audible heartbeat. Postmortem sampling was conducted on macroscopically abnormal tissues or fluid. All samples were collected in a sterile manner, refrigerated, and transferred to the laboratory within 48 h. One human-origin sample (sputum) was submitted for routine TB diagnosis after the patient developed signs of active TB (haemoptysis).

### *Mycobacterium tuberculosis* isolation and identification

All samples were processed at the Institut Pasteur du Cambodge (IPC) during routine diagnostic investigation of cases. Samples underwent NaLC-NaOH decontamination with a final NaOH concentration of 1%^65^. After neutralisation with phosphate buffer (PB) and centrifugation, pellets were resuspended in PB (0.7 ml) then inoculated into a Löwestein-Jensen (LJ) slant and in MGIT liquid culture tubes (Bactec MGIT; BD Microbiology Systems, USA). LJ slants were incubated at 37 °C and monitored weekly for eight weeks; MGIT tubes were loaded into the BACTEC MGIT 960 system for up to 42 days. Any positive cultures were identified as *Mycobacterium tuberculosis* complex (MTBC) using the SD Bioline TB Ag MPT64 rapid test (Standard Diagnostics, Korea). Further identification to distinguish *M. tuberculosis* from other members of the MTBC was undertaken using the HAIN GenoType MTBC assay (Hain Lifescience, Germany).

### Phenotypic antimicrobial susceptibility testing

One isolate from each case was tested for susceptibility to anti-TB drugs, including rifampicin (RIF; 1.0 μg/mL), isoniazid (INH; 0.1 μg/mL), ethambutol (EMB; 5.0 μg/mL), and streptomycin (STM; 1.0 μg/mL) by pDST using the automated BACTEC MGIT 960 (MGIT AST SIRE kit, Becton Dickinson (BD), Sparks, MD, USA)^66^. After routine diagnostic testing all *M. tuberculosis* isolates were stored at -80 °C in the mycobacteriology laboratory at IPC.

### Genotyping

Spoligotyping was performed on one isolate from each case (n = 32) and Mycobacterial Interspersed Repetitive Unit–Variable Number Tandem Repeat (MIRU-VNTR) typing was performed on one isolate from the first 20 cases, as described in detail in Supplementary Material 1.

### Genomic DNA extraction and DNA sequencing

From each of the original isolates (n = 102), a subculture was grown on a LJ medium slant. Three loops of pure colony were harvested into Tris–EDTA buffer pH8 and inactivated by heating to 95 °C for 30 min. Inactivation was confirmed by subjecting a random sample of 34 inactivated samples to culture in MGIT media, with no growth observed. Heat inactivated isolates were stored at -20 °C prior and after shipment at 2–8 °C to Murdoch University, Australia.

Approximately 0.5 ml of heat-inactivated mycobacterial culture was brought to room temperature and centrifuged at 1000 × G for 5 min. After removal of the supernatant, the cell pellet was resuspended in lysis buffer (sodium dodecyl sulphate; 180 μl) and treated with proteinase K (20 mg/ml; 25 μl) and incubated overnight on a thermomixer at 56 °C. After the lysis step, a Qiagen DNeasy Blood and Tissue kit (Qiagen, Germany) was used to extract mycobacterial genomic DNA following the manufacturer’s instructions for the purification of total DNA from animal tissues (Spin-Column protocol). DNA was eluted with 10 mM Tris (50 μl). Library preparation was conducted using a Celero DNA-Seq library preparation kit (NuGEN-Tecan, Switzerland)^67^, while library preparations were sequenced via Illumina NextSeq platform with a high output 2 × 150 kit.

### Bioinformatics and phylogenetics

Short reads were mapped to the H37RV (Genbank accession number NC_000962.2) *M. tuberculosis* reference genome using Stampy version 1.0.17^68^. Repetitive regions were masked alongside four genes containing high levels of artefactual variation (*tuf, rrs, rrl, Rvnt38*)^64,69^. SAMtools mpileup version 0.1.18^70^ was used to call variants using a minimum read depth of 5× and at least one read on each strand. A single nucleotide polymorphism (SNP) threshold was used to define clusters after multi-FASTA alignment, as previously described^64^, whereby any sequence within the defined SNP threshold of another in a cluster was considered part of the same cluster. Python software is available^71^.

Phylogenetic trees were built using PhyML after aligning FASTA files, identifying SNPs across them, and padding the H37Rv reference genome with those SNPs under the assumption that missing data at non-variant positions are wild type. Trees were viewed in a radial layout using Figtree v.1.4.4^72^ and re-drawn for the purpose of the figures. Null calls at variant sites were inspected manually to see if these could provide additional information on direction of transmission.

### Supplementary Information

Supplementary Information.

## Data Availability

The sequences generated and analyzed during this study are available from the National Centre for Biotechnology Information (NCBI) Sequence Read Archive database under BioProject PRJNA1007403. Accession numbers for each isolate are provided in Supplementary Material Table S2.
