# Supplementary material for: Genomic insights into anthropozoonotic tuberculosis in captive sun bears (Helarctos malayanus) and an Asiatic black bear (Ursus thibetanus) in Cambodia
Source: Sci Rep. 2024 Mar 28;14:7343. doi: 10.1038/s41598-024-57318-1 (PMC10973429; doi:10.1038/s41598-024-57318-1)
Supplement: Supplementary file 1 — Supplementary Information. [file 41598_2024_57318_MOESM1_ESM.pdf]

Supplementary Material 1: Genotyping methods and results

Genotyping methods

DNA preparation

M. tuberculosis isolates were re-cultured on LJ medium slants, and a loopful of *M. tuberculosis* colonies suspended in 300 μL of Tris-EDTA buffer (10 mM Tris, 1mM EDTA pH 8.5) and heated for 30 min at 95°C. After centrifugation the supernatant containing bacterial DNA was used as a template for PCR based genotyping techniques.

Spoligotyping

Spoligotyping was performed on one isolate from each case (n=32), following previously described techniques [1]. The DNA of *M. tuberculosis* H37Rv and *M. bovis* BCG were included as positive controls. Spoligotype patterns were coded as a binary number and entered into the international genotyping database SITVIT2 (available at http://www.pasteur-guadeloupe.fr:8081/SITVIT2/) to identify the spoligotype international type (SIT) [2].

Mycobacterial Interspersed Repetitive Unit – Variable Number Tandem Repeat (MIRU-VNTR) typing

MIRU-VNTR typing was performed on one isolate from the first 20 cases, by PCR amplifying 24 loci sets as described by Supply et al. [3]. PCR analyses were carried out manually for each of 24 loci. PCR fragments were separated by electrophoresis using a 1.5% agarose gel. The amplicon size was estimated by comparison with a 100 bp molecular marker. The number of repeats at each locus was calculated using an allele-calling table and used to

create a 24-digit allelic profile for each isolate [3]. The results were analysed using the MIRU-VNTRplus database (available at at www.miru-vntrplus.org/) [4].

Genotyping results

Spoligotyping

The spoligotype patterns of the 32 *M. tuberculosis* isolates (31 bear-origin and 1 human-origin) were compared to the SITVIT2 database and revealed two patterns (Table S1). The most frequent spoligotype pattern, represented by the octal code 777777777413731 and shared by 21 bear origin isolates, belonged to Spoligo-international-type (SIT) 48 in the EAI1-SOM clade of the East African-Indian (EAI) family of Lineage 1 (Indo-Oceanic). The remaining 11 isolates (ten bear-origin and one human-origin) revealed a SIT1 spoligotype pattern, represented by the octal code 000000000003771 and belonging to the Beijing family of Lineage 2 (East Asian). The 11 Beijing family isolates were the same 11 isolates with drug resistance to isoniazid and streptomycin, while all the EAI family isolates were pan-sensitive.

Mycobacterial Interspersed Repetitive Unit – Variable Number Tandem Repeat (MIRU-VNTR) typing

MIRU- VNTR typing of an isolate from each of the first 20 cases (19 bear and one human) revealed three patterns, including two clusters and one unique profile (Table 1). The first cluster contained eight Beijing family isolates (seven bear-origin, one human-origin), and the second cluster contained 11 of 12 bear-origin isolates with the EAI family spoligotype. The single unique MIRU-VNTR profile belonged to case E07's isolate and showed variation in the number of repeats at locus 802 compared to the other isolates in the second cluster.

References

1. Kamerbeek, J., et al. Simultaneous detection and strain differentiation of *Mycobacterium tuberculosis* for diagnosis and epidemiology. *J Clin Microbiol*. **35**, 907-914 https://doi.org/10.1128/jcm.35.4.907-914.1997 (1997).
2. Couvin, D., David, A., Zozio, T. & Rastogi, N. Macro-geographical specificities of the prevailing tuberculosis epidemic as seen through SITVIT2, an updated version of the *Mycobacterium tuberculosis* genotyping database. *Infect Genet Evol*. **72**, 31-43 https://doi.org/10.1016/j.meegid.2018.12.030 (2019).
3. Supply, P., et al. Proposal for standardization of optimized mycobacterial interspersed repetitive unit-variable-number tandem repeat typing of *Mycobacterium tuberculosis. J Clin Microbiol*. **44**, 4498-510 https://doi.org/10.1128/jcm.01392-06 (2006).
4. Weniger, T., Krawczyk, J., Supply, P., Niemann, S. & Harmsen, D. MIRU-VNTRplus: a web tool for polyphasic genotyping of *Mycobacterium tuberculosis* complex bacteria. *Nucleic Acids Res*. **38**, W326-31 https://doi.org/10.1093/nar/gkq351 (2010).

Table S1. Drug susceptibility, spoligotype lineage and Mycobacterial Interspersed Repetitive Unit – Variable Number Tandem Repeat (MIRU-VNTR) type results of *Mycobacterium tuberculosis* isolates from 30 sun bears (*Helarctos malayanus*), one Asiatic black bear (*Ursus thibetanus*) and one human case at the Cambodia Bear Sanctuary, in chronological order by sampling date and including the date of each bear's arrival at the sanctuary and their age at the time of sampling

Case	Species	Age (y)	Arrival date	Sample date	Drug susceptibility STM/INH/ RIF/EMB	Spoligotype lineage (SIT)	MIRU-VNTR numeric code
**Reference**							224313222534236133335522
**B1**	SB	4	Apr 2007	Dec 2009	R/R/S/S	Beijing (SIT1)	244233262544424183353823
**B2**	SB	5	Jan 2006	Jan 2011	R/R/S/S	Beijing (SIT1)	244233262544424183353823
**Hu**	Human	NA	NA	Jul 2011	R/R/S/S	Beijing (SIT1)	244233262544424183353823
**E1**	SB	16	Oct 2011	Dec 2011	S/S/S/S	EAI1-SOM (SIT48)	224734152263245223354613
**E2**	SB	20	Aug 1996	Mar 2014	S/S/S/S	EAI1-SOM (SIT48)	224734152263245223354613
**E3**	SB	20	Dec 2008	Jul 2015	S/S/S/S	EAI1-SOM (SIT48)	224734152263245223354613
**B3**	SB	5	Jun 2011	Feb 2016	R/R/S/S	Beijing (SIT1)	244233262544424183353823
**B4**	SB	6	Aug 2011	Jul 2016	R/R/S/S	Beijing (SIT1)	244233262544424183353823
**E4**	SB	13	Dec 2003	Aug 2016	S/S/S/S	EAI1-SOM (SIT48)	224734152263245223354613
**B5**	SB	5	Oct 2011	Aug 2016	R/R/S/S	Beijing (SIT1)	244233262544424183353823
**E5**	SB	11	Feb 2006	Nov 2016	S/S/S/S	EAI1-SOM (SIT48)	224734152263245223354613
**E6**	SB	21	Jun 2002	Nov 2016	S/S/S/S	EAI1-SOM (SIT48)	224734152263245223354613
**B6**	SB	5	Oct 2012	Feb 2017	R/R/S/S	Beijing (SIT1)	244233262544424183353823
**E7**	SB	18	Oct 2000	Mar 2017	S/S/S/S	EAI1-SOM (SIT48)	2247**4**4152263245223354613
**B7**	SB	5	Nov 2011	Mar 2017	R/R/S/S	Beijing (SIT1)	244233262544424183353823
**E8**	SB	17	Aug 2003	Mar 2017	S/S/S/S	EAI1-SOM (SIT48)	224734152263245223354613
**E9**	SB	22	Oct 2004	Mar 2017	S/S/S/S	EAI1-SOM (SIT48)	224734152263245223354613
**E10**	SB	10	Feb 2007	Mar 2017	S/S/S/S	EAI1-SOM (SIT48)	224734152263245223354613
**E11**	SB	11	Jul 2006	Mar 2017	S/S/S/S	EAI1-SOM (SIT48)	224734152263245223354613
**E12**	SB	11	May 2006	Mar 2017	S/S/S/S	EAI1-SOM (SIT48)	224734152263245223354613
**E13**	SB	24	Aug 1996	Nov 2017	S/S/S/S	EAI1-SOM (SIT48)	Not done
**E14**	SB	23	Aug 2003	Nov 2017	S/S/S/S	EAI1-SOM (SIT48)	Not done
**B8**	SB	8	Jun 2010	Nov 2017	R/R/S/S	Beijing (SIT1)	Not done
**E15**	SB	18	Nov 2001	Dec 2017	S/S/S/S	EAI1-SOM (SIT48)	Not done
**B9**	SB	5	Mar 2013	Feb 2018	R/R/S/S	Beijing (SIT1)	Not done
**B10**	SB	5	Sep 2013	Feb 2018	R/R/S/S	Beijing (SIT1)	Not done
**E16**	SB	23	Nov 2001	Aug 2018	S/S/S/S	EAI1-SOM (SIT48)	Not done
**E17-ABB**	ABB	9	Jul 2010	Nov 2018	S/S/S/S	EAI1-SOM (SIT48)	Not done
**E18**	SB	23	Jun 1999	Nov 2018	S/S/S/S	EAI1-SOM (SIT48)	Not done
**E19**	SB	24	Apr 1999	Dec 2018	S/S/S/S	EAI1-SOM (SIT48)	Not done
**E20**	SB	20	Sep 2005	Dec 2018	S/S/S/S	EAI1-SOM (SIT48)	Not done
**E21**	SB	27	Apr 2005	Mar 2019	S/S/S/S	EAI1-SOM (SIT48)	Not done

SB = sun bear; ABB = Asiatic black bear; NA = not applicable; SIT = Spoligo international type
R = resistant; S = susceptible; STM = streptomycin; INH = isoniazid; RIF = rifampicin; EMB = ethambutol
MIRU loci patterns are in the order 154, 424, 577, 580, 802, 960, 1644, 1955, 2059, 2163b, 2165, 2347, 2401, 2461, 2531, 2687, 2996, 3007, 3171, 3192, 3690, 4052, 4156, 4348

Table S2. Lineage and National Center for Biotechnology Information BioSample accession numbers for 100 successfully sequenced *Mycobacterium tuberculosis* isolates from 30 sun bears (*Helarctos malayanus*), one Asiatic black bear (*Ursus thibetanus*) and one human case at the Cambodia Bear Sanctuary, and including case and isolate references and sample origin

**Case ID**	**Isolate reference^**	**Sample origin**	**Lineage**	**Accession number**
**B1**	B1	Bronchoalveolar lavage	Lineage 2	SAMN37068676
	B1	Lung	Lineage 2	SAMN37068677
	B1	Gastric fluid	Lineage 2	SAMN37068675
**B2**	B2a	Lung	Lineage 2	SAMN37068678
	B2b	Lung	Lineage 2	SAMN37068679
	B2b	Lung	Lineage 2	SAMN37068681
	B2b	Pleural fluid	Lineage 2	SAMN37068680
**Hu**	Hu	Sputum	Lineage 2	SAMN37068682
**B3**	B3a	Tracheal mucous	Lineage 2	SAMN37068688
	B3b	Bronchoalveolar lavage	Lineage 2	SAMN37068689
**B4**	B4a	Bronchoalveolar lavage	Lineage 2	SAMN37068690
	B4a	Wound	Lineage 2	SAMN37068692
	B4a	Wound	Lineage 2	SAMN37068693
	B4a	Oral swab	Lineage 2	SAMN37068691
	B4b	Lung	Lineage 2	SAMN37068694
**B5**	B5a	Lung	Lineage 2	SAMN37068699
	B5b	Cervical lymph node	Lineage 2	SAMN37068698
	B5b	Oral biopsy	Lineage 2	SAMN37068695
**B6**	B6a	Lung	Lineage 2	SAMN37068707
	B6a	Peritoneal fluid	Lineage 2	SAMN37068705
	B6a	Sub-cutaneous tissue	Lineage 2	SAMN37068706
	B6a	Intestinal abscess	Lineage 2	SAMN37068708
**B7**	B7a	Mesenteric lymph node	Lineage 2	SAMN37068713
	B7b	Tongue lesion	Lineage 2	SAMN37068715
	B7b	Small intestine	Lineage 2	SAMN37068714
	B7c	Lung	Lineage 2	SAMN37068716
**B8**	B8	Submandibular lymph node	Lineage 2	SAMN37068736
	B8	Tracheobronchial lymph node	Lineage 2	SAMN37068735
**B9**	B9	Submandibular lymph node	Lineage 2	SAMN37068743
**B10**	B10	Tracheobronchial lymph node	Lineage 2	SAMN37068744
	B10	Mesenteric lymph node	Lineage 2	SAMN37068745
**E1**	E1	Bronchoalveolar lavage	Lineage 1	SAMN37068683
**E2**	E2	Bronchoalveolar lavage	Lineage 1	SAMN37068684
**E3**	E3a	Lung	Lineage 1	SAMN37068686
	E3a	Pleural fluid	Lineage 1	SAMN37068687
	E3b	Lung	Lineage 1	SAMN37068685
**E4**	E4	Bronchoalveolar lavage	Lineage 1	SAMN37068696
	E4	Lung	Lineage 1	SAMN37068697
**E5**	E5	Bronchoalveolar lavage	Lineage 1	SAMN37068700
	E5	Lung	Lineage 1	SAMN37068703
	E5	Faeces	Lineage 1	SAMN37068702
**E6**	E6	Lung	Lineage 1	SAMN37068704
	E6	Faeces	Lineage 1	SAMN37068701
**E7**	E7	Bronchoalveolar lavage	Lineage 1	SAMN37068710
	E7	Lung	Lineage 1	SAMN37068712
	E7	Tracheal mucous	Lineage 1	SAMN37068709
	E7	Pleural fluid	Lineage 1	SAMN37068711
	E7	Faeces	Lineage 1	SAMN37068717
**E8**	E8	Bronchoalveolar lavage	Lineage 1	SAMN37068718
	E8	Lung	Lineage 1	SAMN37068720
	E8	Faeces	Lineage 1	SAMN37068719
**E9**	E9	Lung	Lineage 1	SAMN37068721
**E10**	E10a	Lung	Lineage 1	SAMN37068722
	E10b	Bronchoalveolar lavage	Lineage 1	SAMN37068723
**E11**	E11	Lung	Lineage 1	SAMN37068725
	E11	Faeces	Lineage 1	SAMN37068726
**E12**	E12	Lung	Lineage 1	SAMN37068724
**E13**	E13	Bronchoalveolar lavage	Lineage 1	SAMN37068729
	E13	Lung	Lineage 1	SAMN37068728

	E13	Tarsal joint cartilage	Lineage 1	SAMN37068727
**E14**	E14	Bronchoalveolar lavage	Lineage 1	SAMN37068732
	E14	Lung	Lineage 1	SAMN37068734
	E14	Tracheobronchial lymph node	Lineage 1	SAMN37068730
	E14	Mediastinal lymph node	Lineage 1	SAMN37068733
	E14	Liver	Lineage 1	SAMN37068731
**E15**	E15	Bronchoalveolar lavage	Lineage 1	SAMN37068741
	E15	Lung	Lineage 1	SAMN37068737
	E15	Lung	Lineage 1	SAMN37068738
	E15	Pleural fluid	Lineage 1	SAMN37068742
	E15	Tracheobronchial lymph node	Lineage 1	SAMN37068739
	E15	Mesenteric lymph node	Lineage 1	SAMN37068740
**E16**	E16	Wound	Lineage 1	SAMN37068746
	E16	Wound	Lineage 1	SAMN37068747
	E16	Wound	Lineage 1	SAMN37068748
	E16	Mediastinal lymph node	Lineage 1	SAMN37068749
**E17-ABB**	E17-ABB	Bronchoalveolar lavage	Lineage 1	SAMN37068752
	E17-ABB	Lung	Lineage 1	SAMN37068751
	E17-ABB	Liver	Lineage 1	SAMN37068750
**E18**	E18a	Bronchoalveolar lavage	Lineage 1	SAMN37068757
	E18a	Lung	Lineage 1	SAMN37068754
	E18a	Mediastinal lymph node	Lineage 1	SAMN37068753
	E18a	Tracheobronchial lymph node	Lineage 1	SAMN37068759
	E18a	Mesenteric lymph node	Lineage 1	SAMN37068758
	E18b	Prescapular lymph node	Lineage 1	SAMN37068755
	E18c	Prescapular abscess	Lineage 1	SAMN37068756
**E19**	E19	Bronchoalveolar lavage	Lineage 1	SAMN37068760
	E19	Lung	Lineage 1	SAMN37068762
	E19	Tracheobronchial lymph node	Lineage 1	SAMN37068761
	E19	Mesenteric lymph node	Lineage 1	SAMN37068764
	E19	Peritoneal fluid	Lineage 1	SAMN37068763
**E20**	E20	Lung	Lineage 1	SAMN37068768
	E20	Mediastinal lymph node	Lineage 1	SAMN37068765
	E20	Spleen	Lineage 1	SAMN37068766
	E20	Liver	Lineage 1	SAMN37068767
	E20	Faeces	Lineage 1	SAMN37068769
**E21**	E21	Bronchoalveolar lavage	Lineage 1	SAMN37068770
	E21	Lung	Lineage 1	SAMN37068772
	E21	Submandibular lymph node	Lineage 1	SAMN37068774
	E21	Mediastinal lymph node	Lineage 1	SAMN37068773
	E21	Mesenteric lymph node	Lineage 1	SAMN37068771

^Isolates from each case with no genetic variation are grouped together and referred to by the case ID and a lower-case letter
